# Hydroxychloroquine-Induced Retinopathy in Systemic Lupus Erythematosus: Predictors of Progression Following Drug Discontinuation

**DOI:** 10.21203/rs.3.rs-10488914/v1

**Published:** 2026-08-25

**Authors:** Emily Gutowski, Yasha Modi, Alfredo Gutierrez Velez, Joseph Colcombe, Carol Lee, Jessica Dai, Erin Carter, Juliet Izmirly, Mala Masson, Brooke Cohen, Chung-E Tseng, Amit Saxena, H. Michael Belmont, Jill Buyon, Peter Izmirly

**Affiliations:** NYU Langone Health; NYU Langone Health; NYU Langone Health; NYU Langone Health; NYU Langone Health; NYU Langone Health; NYU Langone Health; NYU Langone Health; NYU Langone Health; NYU Langone Health; NYU Langone Health; NYU Langone Health; NYU Langone Health; NYU Langone Health; NYU Langone Health

**Keywords:** hydroxychloroquine, systemic lupus erythematosus, hydroxychloroquine retinopathy, retinal toxicity

## Abstract

**Background:**

Hydroxychloroquine (HCQ) is a cornerstone of systemic lupus erythematosus (SLE) management. However, the medication can accumulate within organs and cause toxicity including retinopathy. HCQ is usually discontinued once ocular toxicity is diagnosed, but this is not always sufficient to avoid further damage. Whether retinal injury progresses following HCQ cessation, and which patients are at risk, is of great interest. Prior studies examining progression after HCQ discontinuation are sparse and limited by small sample sizes, and none specifically examine patients with SLE. This study aimed to identify predictors of progression of HCQ-induced retinopathy in SLE patients after medication discontinuation.

**Methods:**

We queried the NYU Lupus Cohort to identify patients with documented HCQ-induced retinopathy who discontinued HCQ and had at least one optical coherence tomography (OCT) follow-up to assess for progression. Demographic information, cumulative HCQ dose, duration of therapy, presence of chronic kidney disease or tamoxifen use, SLE disease activity, and ophthalmologic follow-up intervals were collected. Two ophthalmologists independently reviewed OCT and Humphrey visual field data to confirm retinopathy, grade its severity, and determine progression following drug discontinuation. Statistical analysis was performed using Student’s t-test or Wilcoxon rank-sum test for continuous variables and Chi-square or Fisher’s exact test for categorical variables.

**Results:**

Twenty-one patients with confirmed HCQ retinopathy were included. The mean duration of HCQ therapy preceding diagnosis was 16.2 years and the mean cumulative exposure was 1884 g. Retinal toxicity progressed in 6 patients (28.6%) despite HCQ discontinuation. There appeared to be a trend towards a greater likelihood of progression with more severe retinal toxicity at the time of diagnosis. However, there were no differences between progressors and non-progressors with respect to age at diagnosis, duration or cumulative HCQ exposure, SLE disease activity, tamoxifen use, chronic kidney disease, or guideline-concordant care.

**Conclusions:**

Nearly thirty percent of patients with SLE and HCQ retinopathy progressed despite drug discontinuation. No baseline clinical predictors were associated with progression, though there was a descriptive trend toward greater risk with more severe baseline retinopathy. These data emphasize the importance of continued ocular surveillance for progression of toxicity after discontinuation.

## Background

Hydroxychloroquine (HCQ) is a cornerstone of systemic lupus erythematosus (SLE) management. Hydroxychloroquine has myriad benefits in SLE, including reducing flare rates, improving responses to other treatment modalities, and lowering the frequency of thrombotic and cardiovascular events in patients with antiphospholipid antibodies^1^. As a non-immunosuppressive drug, its safety profile is particularly appealing, and side effects are typically mild, such as skin hyperpigmentation and rash^2–4^. However, the medication can accumulate within organs and cause toxicity including cardiomyopathy, neuropathy, and retinopathy^4^.

Yearly ophthalmologic screening in patients on hydroxychloroquine is vitally important to detect retinopathy before it becomes clinically significant^5–8^. Diagnosis of HCQ-retinopathy is achieved using one structural and one functional test, typically OCT and HVF testing, respectively^5,7^. The prevalence of retinal toxicity attributable to hydroxychloroquine is as high as 7.5% in patients after 5 years, and 20% in patients on HCQ over 20 years^6^ although a subsequent study showed the overall risk for hydroxychloroquine retinopathy was 8.6% after 15 years, and most cases were mild^9^. Patients with total exposures exceeding 1000g of HCQ are known to be at higher risk^10^. Additional established risk factors include kidney disease (OR: 2.1) and use of tamoxifen (OR 4.6), increasing age, female sex, CKD stage 3^11^ as well as preexisting maculopathy^5,6,11^. A recent meta-analysis on risk factors for HCQ maculopathy had a pooled cumulative incidence of 0.1% at 5 years, 2.6% at 10 years, and 5.6% at 15 years. Risk factors were: daily dose > 5 mg/kg, CKD, female sex, and Asian vs. White ethnicity^12^.

Hydroxychloroquine is usually discontinued once ocular toxicity is diagnosed, but this is not always sufficient to avoid further damage^5^. The question of whether retinal injury progresses following drug cessation, and which patients are at highest risk, is of great interest. Previous studies have demonstrated that in some cases, if identified early enough and at a mild stage, retinal damage may improve^13–15^, while in others, damage merely stabilizes or even continues to accumulate following drug discontinuation^6,16,17^. Indeed, prior research has found that due to drug accumulation within the retina, severe or late-diagnosed toxicity may progress for up to two years after cessation^6,16^. These studies are limited by small sample sizes and none specifically examine patients with SLE. This study aims to identify predictors of progression of HCQ-induced retinopathy in SLE patients after medication discontinuation. We sought to ascertain the impact of guideline-concordant care on patient outcomes by considering HCQ dose and frequency of ophthalmologic follow-up prior to the retinopathy diagnosis.

## Methods

We queried the medical records of the NYU Lupus Cohort which has been previously described^18^. 1,319 patients with SLE meeting classification criteria and enrolled through June 2025 were assessed to identify those with documented hydroxychloroquine-induced retinopathy diagnoses. Patients were included if they had records available to confirm the onset of retinopathy and at least one ophthalmology follow-up to assess for progression. We queried the NYU Lupus cohort and supplemented the data by chart review for the following: demographic information; cumulative HCQ dose; duration of therapy; presence of chronic kidney disease (CKD) or tamoxifen use (both HCQ toxicity risk factors^6^); Safety of Estrogens in Lupus Erythematosus National Assessment - Systemic Lupus Erythematosus Disease Activity Index Score (SELENA-SLEDAI Hybrid^19–21^) score and Definition of Remission in SLE (DORIS^22^) status preceding HCQ discontinuation; and interval between ophthalmology visits preceding and following their retinopathy diagnosis. A modified framework was used to assess DORIS remission, as physician global assessment (PGA) was not routinely documented at rheumatology visits. Two ophthalmologists independently reviewed patients’ Optical Coherence Tomography (OCT) and Humphrey visual field (HVF) data to definitively assess whether retinopathy was present, grade its severity, and determine whether there was progression following drug discontinuation. Although one patient was treated with chloroquine, the term “hydroxychloroquine” is used throughout this manuscript to refer broadly to antimalarial therapy for simplicity.

Each individual eye was categorized at baseline and follow-up exams into one of four stages of severity by two ophthalmologists who were blinded to the clinical data. This grading was based on a framework established by Lally et al^17^ (Table 1): Stage 0 was designated for patients without any ellipsoid zone (EZ) attenuation or disruption. In Stage 1, eyes showed subtle irregularities in the foveal or parafoveal region of the ellipsoid zone (EZ), though without definitive disruption. Stage 2 was defined by definite, localized abnormalities in the parafovea, on one or both sides of the fovea, restoration of the EZ anterior to the affected area, without foveal EZ disruption. Stage 3 involved extensive EZ damage in the parafoveal region, though with an intact central foveal EZ. Lastly, Stage 4 had disruption of the EZ extending into the foveal region.

For all eyes, foveal-centered spectral-domain OCT (SD-OCT) volume scans previously obtained as part of routine clinical care were reviewed retrospectively. Imaging was acquired on the Heidelberg Spectralis OCT system (Spectralis; Heidelberg Engineering, Heidelberg, Germany) using a macular cube 512 × 128 scan pattern. The entire macular cube was analyzed and reviewed systematically for each patient, and the initial and final stage was judged. Image grading was performed independently by two ophthalmologists-in-training under the guidance of a vitreoretinal specialist. Scans deemed ambiguous were cross-checked and adjudicated jointly. All available scans from every visit were evaluated to assess for progression. Eyes were considered to have progressed if the eye demonstrated objective changes in EZ integrity, either via a change in classification to a higher severity stage, or an increase in the extent of EZ disruption in the central foveal slice as measured with calipers, even if the severity stage remained unchanged.

Patients’ HCQ dosing and ophthalmology follow-up intervals prior to retinopathy diagnosis were used to classify care as guideline-concordant or -discordant. Patients taking less than or equal to the recommended weight-based dose of HCQ and attending annual ophthalmology visits were considered concordant, while those exceeding the established dosing threshold or with follow-up intervals > 1 year were considered discordant. To account for the American Association of Ophthalmology’s 2016 revision of recommended dosing from 6.5mg/kg/day of body weight to 5 mg/kg/day^7^, patients diagnosed with HCQ retinopathy prior to 2017 were categorized using the former threshold, while current guidelines were used for patients diagnosed in 2017 or later.

Statistical analysis was performed using Student’s t-test or Wilcoxon rank-sum test for continuous variables, as appropriate, and Chi-square or Fisher’s exact test for categorical variables. A p-value < 0.05 was considered statistically significant.

## Results

**Figure 1:**
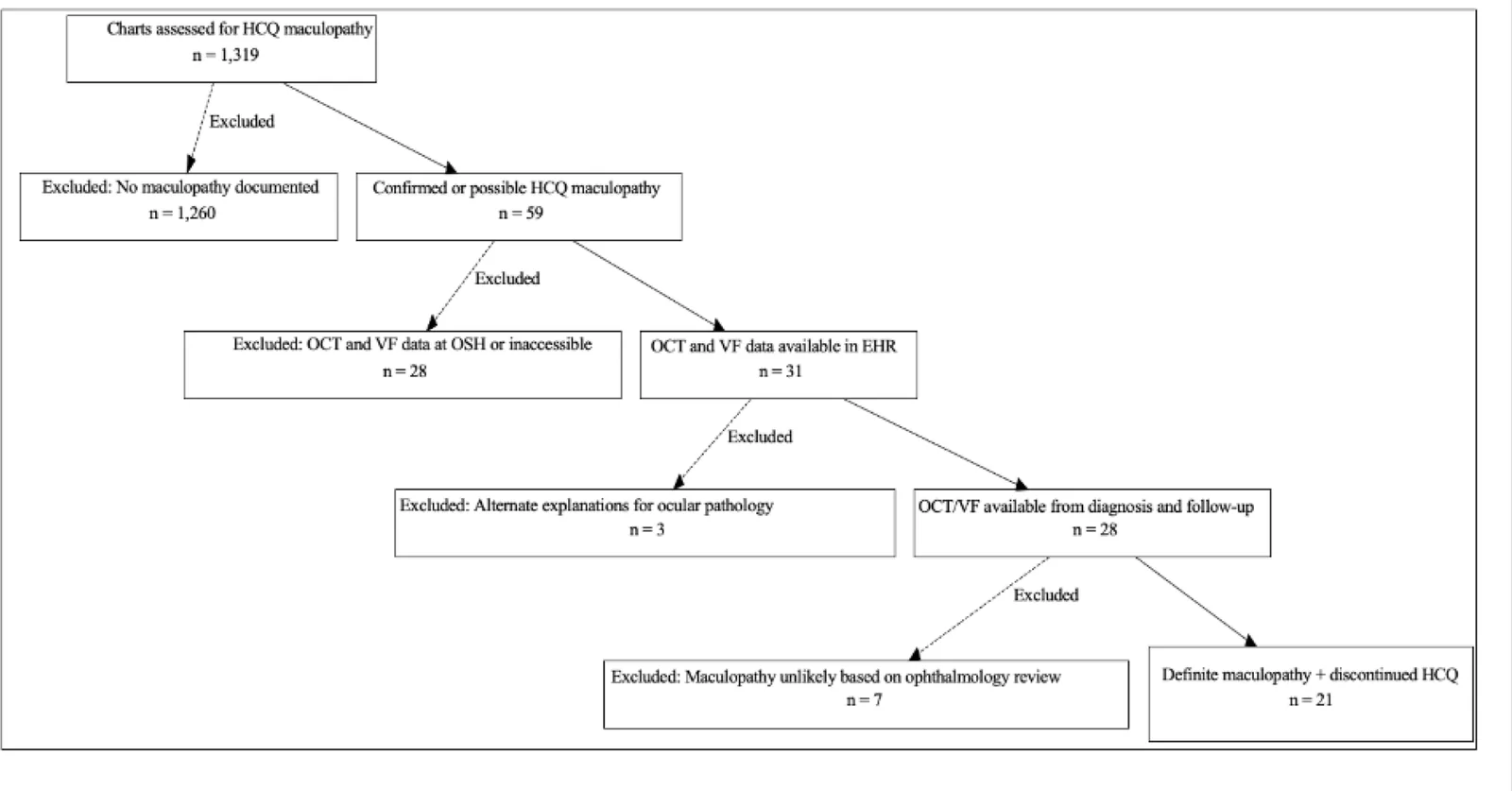
Patient selection flow diagram.

Our initial data query identified 59 out of 1,319 SLE patients who had documented HCQ-induced retinopathy and discontinued the medication. Of these, 31 had medical records available to confirm the onset of retinopathy and at least one ophthalmology follow-up. Upon further review, three patients were excluded due to ocular pathology considered unrelated to HCQ. Ophthalmology review of imaging of the remaining 28 patients excluded seven additional patients due to low likelihood of retinopathy (Fig. 1, Supplemental Table 1).

Of the 21 patients in our final analysis, 20 (95.2%) were women, 7 (33.3%) were White, and 5 (23.8%) were Hispanic (Table 2). The average age at the identification of retinopathy was 53.6 (SD 12.0) years. The mean duration of drug therapy preceding retinopathy was 16.2 (SD 7.6) years and average patient exposure was 1884g (SD 1043.6g) of HCQ over the course of treatment, however this information was limited for one patient whose diagnosis preceded the transition to electronic medical records. Of the 17 patients with available ophthalmology documentation prior to their retinopathy, most (84.2%) were seeing ophthalmology at least yearly, while a smaller proportion (15.8%) had screening lapses preceding their diagnosis.

Documentation from the rheumatology visit preceding retinopathy diagnosis was available for 16 of the 21 patients. Among these patients, the mean SELENA-SLEDAI score at that visit was 1.44 (range, 0–4), indicating minimal to low disease activity. Seven patients (43.8%) were in modified DORIS remission at the time of their retinopathy diagnosis, and nine were not.

In the entire data set of 21 patients with confirmed HCQ retinopathy, six were diagnosed before the 2016 change in guidelines, and 15 were diagnosed after. Only 3 patients of 21 were following guideline-concordant care, 16 were guideline-discordant, and there was insufficient data to determine this information for the remaining 2 patients. Of the 16 guideline-discordant patients, 13 were determined discordant due to doses exceeding the safe recommendation, even after accounting for the change in guidelines in 2016; two were on weight-based doses of HCQ but had lapses in their ophthalmology follow-up (approximately two-year interval between visits); and one was discordant by both HCQ dose and follow-up time (sixteen months). Fourteen of 21 had cumulative doses exceeding 1000g, six did not exceed this number, and one had insufficient records to calculate.

### Progression of retinal toxicity

Retinal toxicity progressed in 6 of 21 patients (28.6%) despite drug discontinuation. Thirteen patients did not progress, and this information was not available for two patients who was diagnosed with maculopathy prior to being in our system but subsequent OCTs, only available starting several years later, showed no progression. Of the six with progression, one was following guideline-concordant care, two were guideline-discordant by HCQ dose but followed with ophthalmology at least yearly, and two had insufficient medical record documentation to determine whether guideline-concordant (Table 3).

The most frequently assigned severity stage at diagnosis was Stage 2 (18 eyes), of which four eyes (22.2%) progressed despite drug discontinuation, 13 (72.2%) did not, and progression was unclear for one eye (for this patient, the first OCT available was from seven years after drug discontinuation, thus progression may have occurred during that time, but subsequent OCTs showed no progression). In 9 eyes the initial severity was Stage 3, of which two eyes (22.2%) progressed, five (55.5%) did not, and progression was unclear for two eyes (this patient’s first available OCT was from ten years after drug discontinuation, though subsequent OCTs showed no progression). All eyes at Stage 3–4 (2 of 2) and Stage 4 (2 of 2) showed clear progression. In contrast, none of the eyes at Stage 1 showed definite progression (0 of 8), with 7 (87.5%) not progressing and progression unclear in one eye for the reasons listed above. No eyes classified as Stage 0 (uncertain, 2 eyes) or Stage 2–3 (1 eye) showed progression (Table 4).

Of the three guideline-concordant patients receiving ≤ 5 mg/kg/day reference body weight (RBW) HCQ and undergoing at least yearly ophthalmologic screening, only one progressed and had been diagnosed with Stage 3 retinal toxicity at diagnosis, progressing to Stage 4 after drug discontinuation (Table 5). This patient did not have previously identified risk factors including kidney disease, tamoxifen use, or concomitant retinal disease.

To better characterize the trajectory of structural changes in the 6 progressors following HCQ discontinuation, we reviewed all available intermediate OCT scans to assess whether progression was gradual, continuous, or plateaued over time. Among the 6 progressors, patterns were heterogeneous. Most demonstrated steady parafoveal EZ/interdigitation zone (IZ) attenuation over multiple years following drug cessation, including conversion from Stage 2 to Stage 3, or from Stage 3 to Stage 4 disease. In one case, unilateral parafoveal progression continued for approximately five years before plateauing, while the contralateral eye remained stable. Other patients showed progression to advanced structural disruption, including subfoveal involvement with EZ/IZ loss or loss of residual foveal islands. In cases with advanced structural disruption at initial diagnosis, subsequent progression was difficult to assess given end-stage disease. In one patient, assessment may have been confounded by concurrent ocular pathology, as subsequent clinical documentation suggested possible retinitis pigmentosa.

All 21 patients continued to see ophthalmology after their retinopathy was diagnosed, with 8 of 21 patients having a follow-up as recently as 2025, 10 in 2024, and 3 in 2022 or 2023. Of the progressors, four of six had an OCT as recently as 2024, while the remaining 2 had their last OCT in 2022. The mean duration of ophthalmology follow-up after retinopathy diagnosis was 6.1 years (SD 4.34).

There was no significant difference between progressors and non-progressors in median age at diagnosis, duration or cumulative HCQ exposure, SELENA-SLEDAI at diagnosis, tamoxifen use, CKD, or receipt of guideline-concordant care (Table 6).

Notably, most patients in this study were evaluated prior to the availability of HCQ level monitoring. Only four of 21 patients had whole blood levels assessed at any time during their exposure to HCQ, with one patient’s levels having been assessed three times during the course of treatment. The mean HCQ whole blood level for these four patients was 1164.85 ng/mL (SD 579.90). As a result of this limited sample size, however, we could not evaluate whether systemic drug levels correlated with risk of retinopathy progression.

## Discussion

In this SLE-specific cohort, nearly one-third of patients demonstrated continued structural progression despite HCQ discontinuation. Although numbers were limited, those with more severe retinal toxicity at the time of diagnosis trended toward a greater likelihood of progression. No statistically significant differences between progressors and non-progressors were identified, including age at diagnosis, duration or cumulative HCQ exposure, disease activity at the time of diagnosis, tamoxifen use, or presence of CKD. While guideline-concordant care was uncommon in this cohort (14%), it did not appear to be protective against retinopathy progression. The recently published 2025 revision of the American Academy of Ophthalmology recommendations further emphasizes the importance of long-term ophthalmologic surveillance in patients on HCQ^8^. Our findings that retinal toxicity may progress despite drug cessation support longitudinal monitoring even after discontinuation of therapy. Our analysis of serial OCT imaging to assess temporal evolution of retinopathy progression found that progression may persist long after HCQ discontinuation, though the rate and extent of deterioration can vary considerably between patients.

Our observations align with prior data that advanced retinopathy is less likely to stabilize following drug discontinuation. A 2014 study by Marmor and Hu assessed the progression of retinal damage in 11 patients with confirmed HCQ retinopathy and found that cases diagnosed prior to retinal pigment epithelial (RPE) damage fared better with preserved foveal architecture and less retinal thinning^23^. In contrast, those diagnosed after RPE damage had already occurred showed progression of retinal damage. A 2019 review by Allahdina et al monitored 22 patients with HCQ retinopathy after discontinuation of the drug, and found that eyes with subtle and localized retinal toxicity largely remained stable and some even demonstrated improvement; however, more severe cases continued to progress^13^. Another review by Ahn et al assessed forty-one patients with HCQ retinopathy and similarly found that only subtle and localized cases remained stable or even improved, while severe cases progressed^15^. Similar findings have been demonstrated in several other studies. Pham and Marmor described 13 patients with different stages of HCQ retinopathy and reported that early and moderate cases stabilized after drug cessation, but severe cases showed continued changes in foveal thickness, EZ line length, visual acuity, and other parameters^16^. They also found that RPE damage present at the time of initial exam was predictive of progressive retinopathy over time. The cohorts in these studies were not limited to those with SLE, and the indication for HCQ was not specified.

The pharmacokinetics of hydroxychloroquine can vary widely between individuals. Whole-blood HCQ concentration is increasingly used to assess patient-specific exposure, and higher levels have been shown to predict later retinopathy^24^. A recent study using the Systemic Lupus Collaborating Clinics (SLICC) Inception Cohort found that HCQ whole blood levels exceeding 1150 ng/mL predicted twice the risk of hydroxychloroquine toxicity^25^. As most of the clinical care for our cohort occurred prior to the widespread availability of this testing, only four patients had levels checked at any time while on hydroxychloroquine.

Our study is limited by its retrospective design and chart review for certain data elements. Its applicability is similarly limited by the fact that it is a single center cohort. Additional limitations include the fact that our sample size overall, as well as the number of patients who progressed despite drug discontinuation, were small limiting statistical analyses. Additionally, data for several patients was limited due to unavailable medical records prior to the transition from paper to an electronic medical system. Another limitation is that patients’ weight may fluctuate over time, and although HCQ dosing was evaluated using the most recent weight available in the medical record, this may not fully capture weight variation throughout the treatment period. Consequently, classification of HCQ dosing as guideline-concordant or -discordant may be imprecise in some cases. Although progression did occur in some cases, it was not always clear from available documentation whether or how visual acuity was impacted.

Lastly, guideline-discordant care may have been influenced by differential access to healthcare, particularly among patients facing socioeconomic barriers. While this study did not systematically assess social determinants of health, the relationship between reduced care access and dosing or screening practices warrants further investigation. Indeed, the period included in this study includes the COVID-19 pandemic, and thus disruptions in access to care as a result of the pandemic may have contributed to lapses in ophthalmologic screening or rheumatologic follow-up during 2020 and early 2021, limiting our ability to fully assess care patterns or progression timelines in certain patients.

This study had several strengths. Our study examined a multi-racial/ethnic large SLE-only cohort, whereas most prior investigations of HCQ retinopathy included patients treated for diverse autoimmune diseases. Restricting the cohort to SLE enhances the clinical relevance of our findings for rheumatology and ophthalmology practice. Our study also is one of the larger studies to look at progression in HCQ maculopathy. This study also uniquely assessed whether patients were following guideline-concordant care based on HCQ dosing and annual ophthalmologic screening, and whether this impacted the risk of progression. Lastly, all cases underwent independent ophthalmologic review of OCT and VF testing to confirm true HCQ retinopathy, and serial OCT data was reviewed longitudinally to characterize not only whether progression occurred, but its pattern and timing.

## Conclusions

Nearly one-third of patients with SLE and hydroxychloroquine retinopathy progressed despite drug discontinuation. This underscores the potential for irreversible disease even after cessation. No baseline clinical predictors were statistically associated with progression, though there appeared to be a trend toward greater risk with more severe baseline retinopathy.

## Supplementary Material

This is a list of supplementary files associated with this preprint. Click to download.

• SupplementalTables.docx

## Figures and Tables

**Table 1:** Staging of severity of HCQ retinopathy^17^.

Stage 0	Unclear staging, uncertain diagnosis
Stage 1	Subtle but not definite disruption of parafoveal EZ
Stage 2	Definite parafoveal EZ disruption, a return of the EZ in the retina anterior to the disruption in the parafoveal region, without foveal EZ disruption
Stage 3	Extensive (on both sides of the fovea) parafoveal EZ disruption but retaining intact foveal EZ in the fovea
Stage 4	EZ disruption involving the fovea

**Table 2:** Demographics and baseline characteristics of patients with confirmed HCQ retinopathy.

Demographics (n = 21)	

Sex: female	20 (95.2%)

Age at retinopathy diagnosis, mean (SD)	53.6 (SD 12.0)

Ethnicity: Hispanic	5 (23.8%)

Race	8 (38.1%)
Asian	7 (33.3%)
White	5 (23.8%)
Black	1 (4.76%)
Other	

Duration of therapy, mean (SD)	16.2y (7.6y)

Cumulative HCQ exposure, mean (SD) (n = 20)	1884.0g (SD 1043.6g)

SELENA-SLEDAI (n = 16)	1.44 (SD 1.58)

Modified DORIS remission (n = 16)	43.8% (7/16)

Additional risk factors	8 (36.4%)
Tamoxifen use	0 (0%)
CKD	6 (28.6%)
Concomitant retinal disease	2 (9.5%)

Following guideline concordant care?	3 (14.3%)
Yes	16 (76.2%)
No	14/16 (68.2%)
Doses exceeding safe recommendation*	2/16 (12.5%)
Lapsed ophthalmology follow-up	2 (9.5%)
Unknown	

Progressed despite discontinuation of HCQ	6 (28.6%)
Guideline-concordant progressors**	1/6 (16.7%)
Guideline-discordant progressors	3/6 (50.0%)
Unknown whether guideline-concordant	2/6 (33.3%)

* >5mg/kg/day using RBW if HCQ if 2016 or later, > 6.5mg/kg/day if before 2016, or > 2.3 mg/kg/day RBW if chloroquine

** Receiving ≤5mg/kg/d RBW HCQ and seeing ophthalmology at least yearly

**Table 3:** Characteristics of progressors despite drug cessation.

Patient #	Eyes with progression	Severity stage at initial diagnosis	Severity stage at most recent follow-up	Age at retinopathy dx (y)	Additional risk factors? (CKD, tamoxifen use, concomitant retinal disease)	Cumulative HCQ exposure (g)	Guideline-concordant care?
1	OS	2 OU	2 OU* (Loss of parafoveal EZ and IZ in OS but no change in stage)	69	No	3577	No: Dosing above recommendation per patient preference. Was seeing ophthalmology every 4 months due to higher dosing.
2	OD	2 OU	3 OD, 2 OS	41	No	Unknown	Unknown: presented after HCQ was discontinued; Unknown prior dosing or follow-up interval.
6	OU	3–4 OU	4 OU	58	No	1387	Unknown: Unknown follow-up intervals, but receiving appropriate dose of medication.
8	OU	4 OU	4 OU* (Loss of EZ and IZ OU but no change in stage as already at end-stage disease)	58	Yes (CKD)	2482	No: >6.5mg/kg/day. Was seeing ophthalmology every 6 months.
18	OU	2 OU	2–3 OU	33	No	1752	No: Dosing above recommendation. Was seeing ophthalmology yearly.
20	OU	3 OU	4 OU	52	No	730	Yes

* Patients had progression (based on EZ attenuation or loss) but no change in severity stage

**Table 4:** Progression by initial stage of retinal toxicity severity. N = 42 for number of eyes.

Severity at diagnosis	Number of eyes	Progressed	Did not progress	Unclear progression
Stage 0 (uncertain)	2	0	2	0
Stage 1	8	0	7	1
Stage 2	18	4	13	1
Stage 2–3	1	0	1	0
Stage 3	9	2	5	2
Stage 3–4	2	2	0	0
Stage 4	2	2	0	0

**Table 5:** Guideline-concordant care patients, receiving ≤5mg/kg/d RBW HCQ and seeing ophthalmology at least yearly (n = 3)

Patient #	Stage of Toxicity at Diagnosis	Progressed?
5	Stage 1	No
7	Stage 3	No
20	Stage 3	Yes

**Table 6:** Variables assessed for association with retinopathy progression despite drug discontinuation using available data only. **Supplemental Tables**

Variable	Progressed (n = 6)	Not Progressed (n = 14)	p-value
**Age at diagnosis**, median	55.0	54.5	0.62
**Duration of therapy**, median (years)	18.0	16.0	1.00
**Cumulative dose**, median (g)	1752	2168	0.85
**SELENA-SLEDAI**, median	0.0 (n = 3)	2.0 (n = 13)	0.88
**Dose ≥ 1000 g**, n (%)	4 (67%)	10 (71%)	1.00
**Guideline concordant**, n (%)	1 (17%)	2 (14%)	1.00
**CKD**, n (%)	2 (33%)	4 (29%)	1.00
**Tamoxifen use**, n (%)	0 (0%)	0 (0%)	–

## Abbreviations

CKD: chronic kidney disease
DORIS: Definition of Remission in SLE
EZ: ellipsoid zone
HCQ: hydroxychloroquine
HVF: Humphrey’s visual field
IZ: interdigitation zone
OCT: Optical Coherence Tomography
OD: Oculus Dexter (right eye)
OR: odds ratio
OS: Oculus Sinister (left eye)
OU: Oculus Uterque (both eyes)
PGA: physician global assessment
RBW: reference body weight
RPE: retinal pigment epithelium
SD: OCT spectral-domain OCT
SELENA: Safety of Estrogens in Lupus Erythematosus National Assessment
SLE: systemic lupus erythematosus
SLEDAI: Systemic Lupus Erythematosus Disease Activity Index 2000
SLICC: Systemic Lupus Collaborating Clinics
